# Exploring everyday work as a dynamic non-event and adaptations to manage safety in intraoperative anaesthesia care: an interview study

**DOI:** 10.1186/s12913-023-09674-3

**Published:** 2023-06-19

**Authors:** Karolina Olin, Charlotte Klinga, Mirjam Ekstedt, Karin Pukk-Härenstam

**Affiliations:** 1 Department of Learning, Informatics, Management and Ethics, Karolinska Institutet, Stockholm, Sweden; 2 Supervisory Centre, Wellbeing Services County of Southwest Finland, Turku, Finland; 3 Stockholm Research and Development Unit for Elderly Persons (FOU Nu), Region Stockholm, Stockholm, Sweden; 4 Department of Health and Caring Sciences, Linnaeus University, Kalmar/Växjö, Sweden; 5 Paediatric Emergency Department, Karolinska University Hospital, Stockholm, Sweden; 6 Department of Women and Children’s Health, Karolinska University Hospital, Stockholm, Sweden

**Keywords:** Resilience, Adaptive capacity, Safety II, Anaesthesia, Complexity, Teamwork

## Abstract

**Background:**

Safety has been described as a dynamic non-event and as constantly present in professionals’ work processes. Investigating management of complex everyday situations may create an opportunity to elucidate safety management. Anaesthesia has been at the frontline of enhancing patient safety – testing and implementing knowledge from other high-reliability industries, such as aviation, in the complex, adaptive system of an operating room. The aim of this study was to explore factors supporting anaesthesia nurses and anaesthesiologists in managing complex everyday situations during intraoperative anaesthesia care processes.

**Methods:**

Individual interviews with anaesthesia nurses (*n* = 9) and anaesthesiologists (*n* = 6) using cognitive task analysis (CTA) on case scenarios from previous prospective, structured observations. The interviews were analysed using the framework method.

**Results:**

During intraoperative anaesthesia care, management of everyday complex situations is sustained through preparedness, support for mindful practices, and monitoring and noticing complex situations and managing them. The prerequisites are created at the organization level. Managers should ensure adequate resources in the form of trained personnel, equipment and time, team and personnel sustainability and early planning of work. Management of complex situations benefits from high-quality teamwork and non-technical skills (NTS), such as communication, leadership and shared situational awareness.

**Conclusion:**

Adequate resources, stability in team compositions and safe boundaries for practice with shared baselines for reoccurring tasks where all viewed as important prerequisites for managing complex everyday work. When and how NTS are used in a specific clinical context depends on having the right organizational prerequisites and a deep expertise of the relevant clinical processes. Methods like CTA can reveal the tacit competence of experienced staff, guide contextualized training in specific contexts and inform the design of safe perioperative work practices, ensuring adequate capacity for adaptation.

**Supplementary Information:**

The online version contains supplementary material available at 10.1186/s12913-023-09674-3.

## Background

In the last decade, the field of safety research has shifted focus from analysing adverse events and errors to understanding how teams and organizations can perform critical tasks and keep processes operational in the face of variations, disruptions and unplanned events [1]. Resilience engineering focuses on understanding and increasing adaptive capacity, providing insight into how care quality and safety arise from multiple interacting factors [2]. Safety has been described as a dynamic non-event [3] and as something that is constantly present in professionals’ work processes. Though resilience is not directly observable, it may be possible to identify its emergence in everyday work, where organizations, teams and professionals manage complex situations through anticipating, monitoring, responding and learning [4, 5]. Resilience engineering started as a theoretical model, highlighted with clinical examples, but there are now a number of studies calling for more rigorous qualitative research, linking the concepts of resilience engineering to concrete strategies for everyday practice among teams and managers [6–8].

It has been suggested that one important way of doing this is through the study of successful performance in everyday situations where professionals interact with each other, technology and the organization to perform complex tasks – i.e., *complex everyday situations* [9]. The characteristics of a complex task, in contrast to a simple task, derive from a system where its components have multiple and dynamic interdependencies [10, 11], making it difficult to predict how the system components will interact in response to a given situation [12]. This implies that each system actor’s view is quite limited – and the faster decisions must be made, the more unpredictable consequences of actions are [13]. Official processes and protocols often reflect the work methods intended to meet the demands at the frontline (WAI – work as imagined). Because of the complex nature of the health care system, with continuous variations and interdependence, the work performed – or work as done (WAD) – is never completely aligned with WAI. As a consequence, resilient performance is dependent on the adaptive capacity of frontline staff managing both the gap between WAI and reality and the challenges that emerge when everyday work is done in a complex adaptive system. This capacity relies on adaptations that professionals perform as a part of everyday work [2, 5, 6].

Anaesthesia teams work in the complex and adaptive system of an operating room (OR) [14], where the intraoperative care process is managed within the anaesthesia team and impacted by the actions of the surgical team [15]. Work during intraoperative anaesthesia has been defined as a combination of clinically and cognitively demanding tasks, paired with high requirements on vigilance and the ability to adjust work based on unexpected developments [8]. Intraoperative anaesthesia work consists of phases with different aims, tasks and intensity, requiring the anaesthesia team’s constant, active presence [16]. Induction was found to be the most intensive phase (73 steps) in the anaesthesiologists’ work process, with errors occurring for example in collecting and preparing medication. Maintenance involved fewer tasks (16 steps), but was prone to errors of a different nature, for example missing relevant information and therefore not acting in time on developments in the patient or procedure status. Solutions for safety management differed between phases. Crosschecks were suggested during induction, whereas systematic checks were recommended during maintenance [17]. In registered nurse anaesthetists’ (RNAs’) intraoperative work, the task frequency, multitasking and interruptions often increased during certain phases, such as anaesthesia induction, preparation for anaesthesia maintenance and extubation. By interpreting multitasking and interruptions as a sign of adaptive capacity, instead of as a threat, they can be explored in regard to how and when resilience emerges [16].

At the individual and team levels, intraoperative patient safety in anaesthesia is also supported by non-technical skills (NTS), defined as ‘the cognitive, social and personal resource skills that complement technical skills, and contribute to safe and efficient care’ [18]. NTS include elements of situational awareness, decision-making, task management and teamwork [19], which have been investigated among anaesthesiologists [20], nurse anaesthetists [21] and anaesthesia assistants [22]. NTS have been linked to certain aspects of resilience, such as adaptive coordination. It has been identified as a feature of high-performing anaesthesia teams and it has been suggested that adaptive capacity may support management of complex and critical tasks in anaesthesia, such as extubating [23]. When facing nonroutine events or when moving from one phase to the next in the anaesthesia work process, the anaesthesia teams showed an increase in task management and adaptation to changing situational demands [24, 25]. Adaptive coordination requires continually appraising the dynamic environment, identifying and defining points of coordination between team members, and making coordination explicit. Exploration of coordination patterns in relation to tasks has revealed that certain patterns may be more effective than others. In addition, task analysis has made it possible to link drivers for action and adaptive behaviours to specific tasks [26].

Thus, insight into prerequisites for successful task management, NTS and the importance of adaptive coordination is growing. However, little is known about the underlying organizational and situational circumstances that trigger and support adaptive coordination and we have only begun to explore the effects that variability in adaptation of NTS have on safety [5, 27]. Such knowledge may contribute to a deeper understanding of how to design care systems for safe clinical practice [1, 14, 26].

In our earlier work, the nature of and challenges to task management during anaesthesia were explored. The aim of this study was to explore factors supporting the anaesthesia team in managing complex everyday situations during intraoperative anaesthesia. The research questions were:

1. How do anaesthesia teams detect and manage complexity during the intraoperative care process?
2. What cognitive strategies do members of anaesthesia teams use to sustain safety in complex everyday situations?
3. From the perspective of clinical experts – what circumstances support or disrupt adaptive capacity in anaesthesia teams?

## Methods

### Study design

An exploratory research design was applied based on data from individual interviews with anaesthesia nurses and anaesthesiologists. In Finland, anaesthesia care is provided by teams consisting of anaesthesia nurses and anaesthesiologists. Anaesthesia nurses assist anaesthesiologists and have the competence to prepare and maintain anaesthesia and monitor anaesthetized patients [28]. An anaesthesiologist may deliver anaesthesia throughout a procedure or delegate this task to an anaesthesia nurse. However, the anaesthesiologist retains personal and professional responsibility for the anaesthetic procedure at all times, even if an anaesthesia nurse is performing most of the work individually [15]. The participants were purposively recruited to capture the perspectives and experiences of all anaesthesia professionals working in the OR context [29].

The framework method [30] was used in the analysis of the qualitative data. The method was chosen as it enables addressing contextual, evaluative and strategic research questions [31]. It is used to identify commonalities and differences in qualitative data and enables drawing descriptive and explanatory inferences clustered around themes. It also enables an approach where a researcher’s interpretations are made transparent through series of interconnected stages [32]. Access to original transcripts and textual data allows others to assess and judge the findings, supporting transparency [33]. A defining feature is the matrix output, which provides a structure into which the researchers can systematically reduce the data. The framework is not aligned with any particular epistemological, philosophical or theoretical approach, nor does it have any allegiance to either deductive or inductive thematic analysis – instead, it incorporates both [30]. During the study, the COVID-19 pandemic prevented close contact between the researchers in this study. The framework method has the additional advantage of supporting collaboration between multi-disciplinary researchers working with large, cross-sectional and descriptive datasets on different sites.

### Setting and study participants

Data collection was conducted in January–February 2020 in Finland, at the anaesthesia departments of three hospitals. In order to prevent single-site bias, the participants were recruited from one university hospital and two county hospitals. The anaesthesia departments in question treated patients in multiple specialities, and provided care to both adult and paediatric patients.

Eligible participants were experienced anaesthesiologists and anaesthesia nurses, who were working clinically in the OR context during the study period. Potential participants were identified with the support of department managers, who had knowledge on the availability of the professionals with the longest clinical experience in the department. Two to three weeks before the interviews, participants were sent a letter with information on the study, on participation being voluntary and on practical arrangements, as well as the researcher’s contact information and a consent form [34]. The sample size of 15 interviews – nine with anaesthesia nurses and six with anaesthesiologists – was chosen based on an information power calculation [35]. The adequacy of the sample size was also assessed during the interviews through preliminary analysis of the data, looking for signs of saturation [36].

### Designing interviews with cognitive task analysis

In order to capture the cognitive skills and strategies underlying the management of both everyday and emergent complex situations, interviews were constructed based on cognitive task analysis (CTA). CTA is an extension of traditional task analysis, often resulting in a description of the performance objectives, equipment, conceptual and procedural knowledge and performance standards used by experts when they perform a task. In addition, CTA provides system design information on how to promote safe patient care [37–40]. Instead of asking experts to recall a critical incident, as in the critical decision method [41], experts are prompted to reflect on everyday work by observing another expert or to reflect on the scenario of a normal situation.

**Fig. 1:**
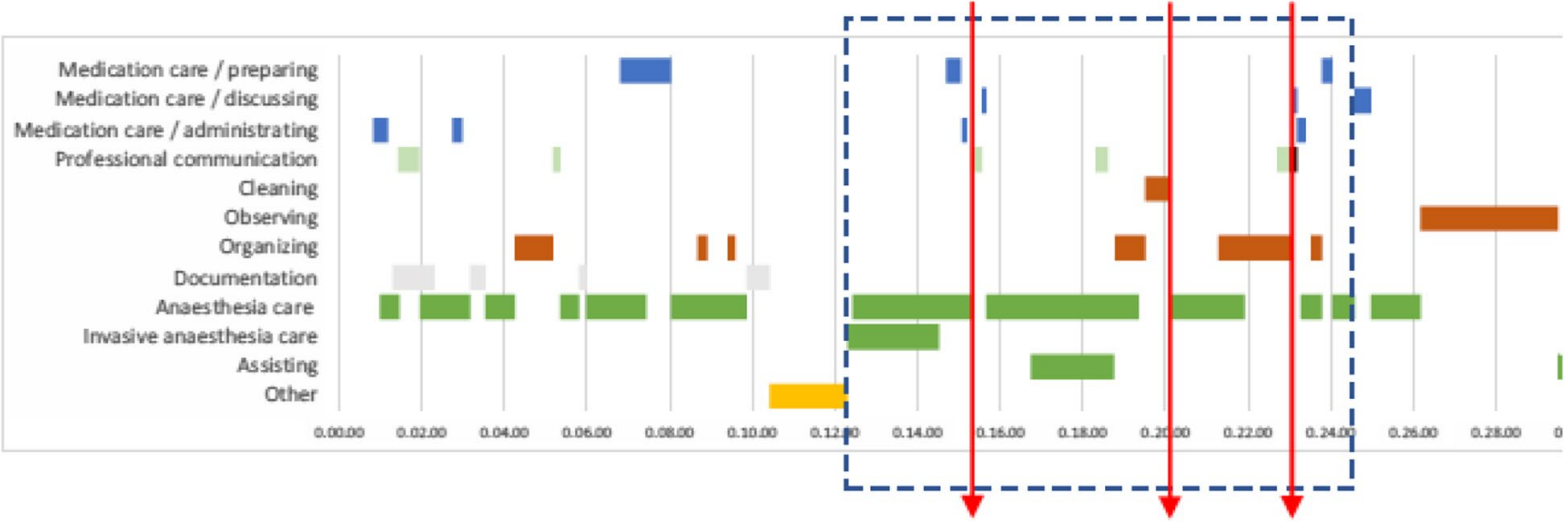
Gantt chart of RNAs’ tasks, multitasking and interruptions during induction, based on previous observations [14]

In this study, the experts were asked to reflect on complex everyday situations visualized through case scenarios. These case scenarios described complex situations in intraoperative work processes and were created specifically for this study, based on data from previous observation studies conducted by members of the research group [14, 16]. Situations with high task frequency, multitasking and interruptions, such as intubation, were included. Gantt charts were created with task categories on the vertical axis and time intervals on the horizontal axis. The resulting charts served as visual representations of two possible scenarios: one expected complex situation (induction, Fig. 1) and one unexpected complex situation during anaesthesia maintenance.

The interview guide, consisting of open-ended questions, was based on previous studies applying CTA [42, 43] (Additional file 1). Pilot interviews were conducted with individuals from both professions, with the aim to adjust the interview guide based on their feedback. The study protocol for the CTA is provided as an Additional file (Additional file 2).

### Data collection

Data were collected by one researcher (KO) through individual scheduled interviews, held in an undisturbed and quiet location at each hospital. At the beginning of each session, the researcher gave a brief presentation of the study, its aim and a declaration of participant confidentiality [44]. The interviews started with a walkthrough of two case scenarios, one illustrating a planned induction and the other an unexpected complex situation during anaesthesia maintenance.

The participants were asked to reflect upon and share experiences from similar situations in their own work. They were then asked to reflect on the cases guided by the questions in the interview guide (Additional file 1, Table 1). Broad descriptions were allowed, in order to capture areas not covered by the original questionnaire. Saturation was reached during the final interviews.

**Table 1:** Example of the use of the interview guide

Walkthrough of the visualization of scenarios using Gantt charts ▪ Planned complex situation with high task intensity, multitasking and interruptions ▪ Emergent, unexpected complex situation with high task intensity, multitasking and interruptions	
Researcher	Can you identify a similar situation at your work? (Question 1)
Participant	*Yes, of course, a routine intubation is … and if especially difficulties arise, it’s a situation where you may have to, like, simultaneously consider multiple options. And like … during maintenance of course a sudden bleeding is a typical, where you have to react … fast. As well as some arrythmias, where the circulation collapses … But they aren’t that common*
Researcher	What thoughts or feelings arise based on the case? (Question 2)
Participant	*Well … It’s a bit difficult to … Of course there’s like … You try to calm the situation down and the first thing on my mind is to list the next steps in my mind, one by one, to execute, so that the aim is achieved. (Covers question 3)*
Researcher	What would help you to handle the situation? (Question 4)
Participant	*Oh yes, if you have an experienced anaesthesia nurse as a partner, who has already an idea of what is going to happen next, it makes the reaction much faster … managing the situation. Of course, if there are interruptions and other disturbances, like phone calls, yes, they make management more challenging. (Covers question 5)*
Researcher	Could you explain this further? (A probing question)
Participant	*My aim is to, like, shut out the external environment and focus on the situation at hand, critical tasks. When the situation is in control, I can open up to additional information which isn’t urgent at that moment* *(Continues)*

### Data analysis

The interviews were audio-recorded and transcribed verbatim. The transcribed data were analysed using the framework method [30]. In this study, the analysis began with an inductive approach, where the researchers familiarized themselves with the transcribed data by reading through them multiple times. During the process of unrestricted coding, conceptual or descriptive labels were assigned to excerpts of raw data. All codes were organized in an Excel sheet, creating a framework method matrix. During abstraction, general descriptions of the research topic were produced through generating subcategories based on the contents of the codes. Each subcategory was named using words characteristic of their contents. Subcategories with similar events and incidents were grouped into categories and categories were grouped into themes (Table 2). The abstraction process was continued as far as it was considered reasonable and/or possible. Any text that could not be indexed within the initial coding scheme was given a new code, meaning that the analysis process included both inductive and deductive elements. The findings are presented as descriptive evidence, reflecting the perceptions of both the professions included [30].

**Table 2:** An example of the abstraction process from codes to themes

Codes	Subcategory	Category	Theme
‘However, here I noticed very quickly that things were always done in the same way. It is like an unwritten law among the personnel, in the beginning, it was very annoying, when I would have liked to do stuff in my own way … but standardization is so much safer …’ (Anaesthesiologist) ‘Well, first we have the routines, it is like the foundation on which the rest is built. You have certain routines and almost anything can happen, but when you have the routines, you can always build on them.’ (Anaesthesia nurse)	Processes and routines described and documented	Standardization of processes and work environment	Organizational prerequisites
‘It is like a jungle of impulses, where you need to navigate. I kind of filter the impulses not meant for me, which are not relevant to my work. They can be clinical signs from the patient, signals from the monitor, with or without the alarm sounding, and quite often communication. So … information which can affect my patient, which is my responsibility, that’s what I try to filter then (Anaesthesiologist) ‘You need to be alert the whole time, you need to absorb a lot of information – from the environment and you have to sort and separate it … some part of it can be completely vital information and can be essential in your work.’ (Anaesthesia nurse)	Filtering relevant information from multiple data sources and senses	Monitoring the patient may provide clues regarding a change	Anaesthesia team members’ strategies

Once the first interview was conducted, two researchers independently analysed the interview transcript using the framework method [30]. The codes and categories created were compared side by side in the framework method Excel sheet, indicating high similarities between the researchers even though only one of them (KO) had profound context knowledge and the other (CK) had no previous experience from the anaesthesia context or an OR. However, the latter had profound experience of qualitative analysis. The resulting two analytical frameworks were found to be highly similar, reflecting credibility [29]. The final codes were organized into subcategories, which were grouped into categories based on similarities in the included concepts. This was discussed by all researchers involved in the study (KO, CK, KPH). The same applied to the next step, in which themes containing several categories were developed.

## Results

### Sample characteristics

In total, 15 professionals participated in the interviews The median length of the interviews was 31 min and 2 s, with an interquartile range (IQR) of 6 min 36 s (Table 3).

**Table 3:** Participant demographics and duration of interviews

Participant	ID (*n* = 15)	Work experience (all), years	Work experience (this unit), years	Duration of interview, (min:sec)
Anaesthesia nurse	1	20	10	29:59
	2	32	30	28:22
	3	30	29	26:34
	4	16	16	31:02
	5	32	32	53:38
	6	22	20	31:04
	7	24	24	30:03
	8	21	19	23:49
	9	30	30	34:09
Mean		25.5 (16–32)	23.3 (10–32) Median	30:03 (IQR 5:09)
Anaesthesiologist	1	14	12	34:00
	2	8	6	26:34
	3	15	13	34:00
	4	12	7	49:32
	5	22	8	35:12
	6	7	4	26:36
Mean		13 (7–22)	8.3 (4–13) Median	34:00 (IQR 8:36)
Total Mean		20.3 (7–32)	17.3 (4–32) Median	31:02 (IQR 6:36)

Both anaesthesia nurses and anaesthesiologists described team members’ strategies and organizational prerequisites for sustaining safety during complex everyday situations (Table 4, Additional file 3).

**Table 4:** Anaesthesia team members’ strategies and organizational prerequisites for sustaining safety during both expected and emergent complex everyday situations within the intraoperative anaesthesia work process

Subcategory	Category	Theme
Planning for anaesthesia during the preceding day Preparing for possible emergencies Using mental models to anticipate events Deep understanding of both anaesthesia and the surgical process	Being prepared	Anaesthesia team members’ strategies
Creating a peaceful atmosphere for the patient Keeping noise levels low, planning the usage of phones and when to interrupt a colleague Using memory aids to focus on the primary task	Supporting mindful practices	
Working adaptively inside the safe boundaries Monitoring the patient may provide clues regarding a change Observing the mood and actions of the surgical team	Monitoring and noticing complex situations	
Prioritizing, knowing the next steps and testing alternative solutions calmly, without delay Clear and undivided leadership Open, timely and honest communication A timeout after a complex situation for checks and feedback	Managing complex situations	
Adequate number of personnel and stable teams Simulation training promotes the ability to react and adapt The personal wellbeing of the anaesthesia professionals Performance and time pressures should be manageable	Enabling adequate levels of resources and competence	Organizational prerequisites
Standardization of processes and work environment Suitable and functional electronic patient records, equipment and appliances	Ensuring an optimal work environment	

### Anaesthesia team members’ strategies

Strategies supporting the management of complex everyday situations – both expected and unexpected – varied between different stages of the anaesthesia process. They included preparing, supporting mindful practices, and monitoring and noticing complex situations and managing them.

#### Being prepared

Planning anaesthesia and providing team members with lists of procedures well in advance, preferably the day before, gave the team the ability to prepare for and anticipate future events. This was considered to be of increasing importance, because patients are older and have more morbidities than in the past.

*We often discuss the day before with the anaesthesiologists, how to prepare for the anaesthesia, what is needed. (Anaesthesia nurse 6)*

The participants emphasized that preparation of anaesthesia takes time, and that this is crucial, for instance when anticipating and preparing a difficult airway with adequate equipment and medication. Also, if a team member was new, the preparation phase was a good opportunity to get to know his/her mental models in relation to anaesthesia care.

*You know how to prepare, maybe with equipment and medications, you know what to take with you … so you don’t have to leave to fetch it, when you should already have started using it. (Anaesthesia nurse 3)*

The participants described how the anaesthesiologist, if he/she arrived during anaesthesia maintenance, could be working through mental models in his/her mind before ‘going in’, anticipating what could be waiting. Cues on the status would be available from the anaesthesia nurses’ tone of voice during reports or phone calls, so relevant information from multiple sources needed to be filtered by the anaesthesiologist. One participant described it as follows:

*Every time I go into the OR, I’m thinking about the phase of the surgical procedure: What should the patient’s status look like and are we starting or in the middle of maintenance … And what has happened in the surgical procedure … or does it have an effect on the patient’s … the situation at hand. (Anaesthesiologist 5)*

Deep understanding of both anaesthesia and surgical processes allowed team members to comprehend the normal rhythm and identify any deviations, so they could anticipate challenges and prioritize tasks if needed. Thus, having performed processes many times made adaptations and preventive risk management easier.

*The anaesthesia nurse anticipates so much, it is almost like telepathic with an experienced team member. So when I have looked there (into a patient’s airway during intubation) for a second and a half, he/she already suggests: ‘Should I put pressure on it (the trachea)?’, it’s so seamless … And the tube is exactly where you need it, so that your hand doesn’t have to do this … (waving an arm to the side). (Anaesthesiologist 3)*

#### Supporting mindful practices

The participants highlighted that meetings with the patient should be conducted without either party feeling rushed. Informing the patient about possible sensations during anaesthesia (e.g., local anaesthesia) could help the patient participate in keeping up situational awareness. This was described by one participant in the following way:

*Maybe with the patients on local anaesthesia, that they are informed from the start, that we discuss with them, I think that’s the most important thing. We don’t mention the surgical risks, but anaesthesia-related ones, definitely, so that the patient doesn’t have to be surprised about their feelings, so there won’t be a panic reaction … You can see quite quickly what kind of a person the patient is. (Anaesthesia nurse 3)*

Keeping noise and music levels low from the beginning of the anaesthesia was mentioned as contributing to keeping focus. Calming things down ahead of intubation or in case of an emergent complex situation was emphasized as an aid in situation management. Phone use was given as an example of an event that could be planned beforehand, especially ahead of critical phases. The importance of allowing anaesthesia nurses to focus when a critical task was at hand was underscored by the participants. In addition, knowing when to interrupt a colleague – whether an anaesthesia nurse, an anaesthesiologist or a member of a surgical team – was mentioned as important, as well as asking students to wait with any questions until a complex situation was over. One participant described the challenge of finding the right time for questions as follows:

*It is always a somewhat challenging situation, knowing when to present a question to the operating surgeon, like if they are in a tight spot, I don’t want to disturb them until it’s under control. (Anaesthesiologist 2)*

Anaesthesia nurses mentioned the use of memory aids as a facilitating factor to actively retain their focus on any important primary task. In a complex situation, anaesthesia nurses found it essential to be constantly vigilant, so that nothing would be forgotten, for instance by checking and double-checking lookalike/soundalike medication.

*You keep your hand on the sevoflurane lid so that you don’t start with something else, until you reach the three percent, and then you close it. (Anaesthesia nurse 6)*

#### Monitoring and noticing complex situations

Anaesthesia nurses described how anaesthesiologists created safe boundaries, inside which they could adaptively ensure stable and effective anaesthesia for the patient.

*We have certain limits for vital parameters and my aim is to keep them within those boundaries. Of course, if a patient is bleeding heavily, I may let the blood pressure drop intentionally, but will raise it back to what it was once the bleeding is under control. (Anaesthesia nurse 5)*

The participants were of the opinion that an anaesthesia team must be aware that something unexpected may happen at any time. Anaesthesia nurses described how they worked through a mental model, monitoring the patient at all times and considering every observed vital parameter. A patient needs to be monitored continuously, both as regards vitals and as a whole. The participants emphasized that changes in a patient’s vitals, identified through continuous monitoring, might indicate problems.

*You have this thought pattern in your mind and you go through it, you check the pulse, blood pressure, saturation, what they look like, end tidal, what is coming out from the patient, and just like that – you go through the pattern, like blood pressure related to the medications the patient has been given, and you check that there has been no bleeding, you question the validity of every finding all the time … That’s partly risk management, keeping yourself under continuous scrutiny. (Anaesthesia nurse 6)*

The participants stated that actively observing the behaviour and communication of the surgical team and sensing changes in their mood could aid identification of possible challenges in anaesthesia care, for example during massive blood loss. Being one step ahead was made possible by observing the surgical process and its rhythm and raising awareness based on anything unusual. This required receiving and filtering relevant information from multiple data sources and through various senses (hearing, sight, smell).

*It depends on both visuals and audio input, you can hear it … if there is haste on the side of the operation room nurse, circulating nurse or surgeon, what kind of communication there is. And if their movements are very abrupt and quick. (Anaesthesiologist 1)*

#### Managing complex situations

Both professions described the importance of prioritization in complex situations. When encountering a complex situation, anaesthesiologists described active problem-solving through testing alternative solutions without delay. In addition, anaesthesia nurses discussed the importance of knowing what they needed in order to achieve the next step. The professionals’ mood was also mentioned. It was considered bad to hurry. Being focused, efficient and calm aided management.

*You must remember to always proceed to the next step, you have to keep trying again and not getting stuck in a situation. (Anaesthesiologist 3)*

Anaesthesia nurses described that they preferred to have one anaesthesiologist leading the work in complex situations. Moreover, clear delegation of responsibilities and clear instructions were underlined as important, especially if a team member was new. Being able to ask for multi-professional help was seen as an asset. However, the participants said that only the relevant people were wanted at the anaesthesia team’s side during a complex situation. In very hectic moments, an anaesthesia nurse would prefer to take responsibility for her own tasks, without helpful hands intruding, as described here by one participant:

*If it is a very complex situation, I try to coordinate, ‘you can write’ and ‘take care of the blood order’, so that I can take care of the rest on my own, thank you. Don’t mess with my anaesthesia. (Anaesthesia nurse 2)*

Both professions emphasized the importance of open, timely and honest communication between anaesthesia professionals and the surgical team. Surgeons could for example provide information in advance about changes that might require anticipatory actions in anaesthesia. Closed-loop communication was preferred, especially if a situation was emergent. In addition, anaesthesia nurses stated that team members should use the same language in all communication during anaesthesia.

*Orders from the firing control must be unambiguous and they can never fall short. So you have to repeat them back, it is like a clear protocol – without that, it’s hard to operate. Like, then the situation is enlightened and opens up to others as well. Like, they’re executing that task now, so I can do this at the same time. (Anaesthesia nurse 6)*

Both professions described it as good practice to perform a quick debriefing after an emergent situation and its management, including discussing patient status, intravenous lines, positioning, etc. Giving and receiving honest feedback within the team, also after successful operations, was highlighted as crucial by all participants. A quick debriefing after an emergent situation could cover how and why the situation was a success.

*After the complex situation, I can quickly walk through the situation with the new anaesthesiologist, and discuss what happened, and document possible identified risks in the patient journal too. (Anaesthesiologist 5)*

### Organizational prerequisites

Organizational prerequisites included adequate levels of resources and competence, in addition to an optimal work environment.

#### Enabling adequate levels of resources and competence

Both anaesthesia nurses and anaesthesiologists described how an adequate number of personnel was the foundation for safe anaesthesia care. Committed staff members supported the sustainment of high levels of competence. Stable team composition and having the opportunity to focus on limited specialties contributed to cumulation and construction of expertise. In addition, in-depth competence and professional skills created the capacity for development.

*The challenge is that not all anaesthesia nurses have the time to focus on paediatric anaesthesia. They circulate all over the unit, they know bits and pieces of everything, but it leads to a situation where no one is actually all that good anymore. (Anaesthesiologist 2)*

Anaesthesiologists mentioned the importance of participating in team training such as simulations, to build the individual capacity to react and adapt. The participants underlined that teams should have frequent possibilities of simulation training with all team members. Both professions identified adequate orientation as a prerequisite for forming routines and stated that adequate training ensured competence. Multi-professional training was also pointed out as necessary when using new equipment.

*And the way I see it, it requires training, not sitting through a lecture, because that may not lead to a good outcome. But a suitable amount of team training and development, so that you are left with good practical skills. And it is of paramount importance that you participate with your own team. (Anaesthesiologist 5)*

The participants underlined the importance of feeling rested and having an adequate level of alertness, which enabled them to deal with interruptions and react swiftly. They said that having a culture that supported wellbeing and success created a positive atmosphere, which in turn supported management of complex situations.

*It has happened to me, as well, a couple of times, I have been too tired at the end of a night shift to think properly … so … a couple of times I have been very, very close to making a serious mistake. (Anaesthesiologist 1)*

It was highlighted by the participants that performance pressures should be manageable. Having an overly tightly packed schedule was mentioned as causing unwanted and suboptimal multitasking.

*Like, we have these gastric bypass operations three in a row, they all have an insane amount of drugs, all of them, more than in any other and all drawn in syringes. So you have to draw the medication for the next anaesthesia during the one you are managing. I mean, I can observe the monitors at the same time, but it feels far from optimal. (Anaesthesia nurse 5)*

#### Ensuring an optimal work environment

Both professions stated that an organization should have standardized protocols. Processes and protocols should be described and documented, because working adaptively was found to be easier when done inside safe boundaries and when clear instructions were given. In addition, a familiar and standardized work environment and placement of equipment and medications was emphasized as facilitating management of complex situations.

*We had no room for mistakes in paediatrics. We thought very carefully about what we did, what kind of routines we had, and we did everything in the same way, like a train on rails. Of course there were patient-specific variations every time, but standardization was the foundation we built on. (Anaesthesia nurse 8)*

Anaesthesiologists highlighted the importance of suitable and functional electronic patient records for management of complex situations. In addition, it was stated that quality should be the main criterion in procurement procedures for new equipment and appliances – so equipment was reliable and did not fail in a complex situation.

*A lot of time and effort is wasted in situations when we have received unsuitable equipment through procurement procedures. Starting with ophthalmologists’ ‘suction coagulators’, in which either suction or coagulation works, and intravenous cannulas which are really difficult to insert. (Anaesthesiologist 5)*

## Discussion

**Fig. 2:**
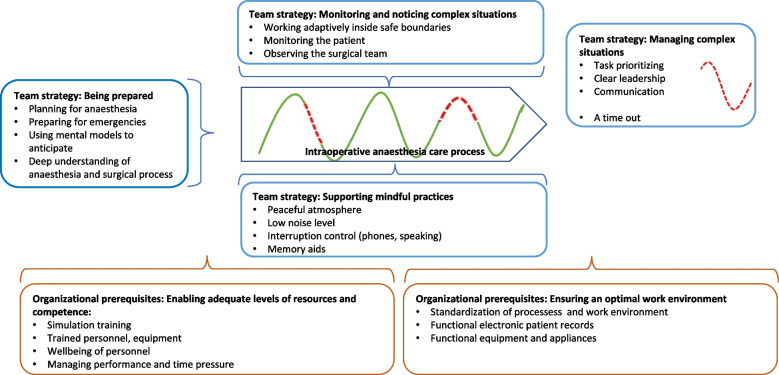
Organisational prerequisites and anaesthesia teams’ strategies in managing safety in the intraoperative anaesthesia care process

As resilience is not directly observable as a phenomenon, the research questions were explored using a combination of scenarios constructed from structured, prospective observations (WAD) and individual interviews using CTA. The participants provided rich examples of strategies used by anaesthesia teams to detect and manage complex everyday situations that required adaptation as well as elucidating organizational factors reflecting and supporting resilience (Fig. 2).

### Strategies

To ensure *being prepared* for both unexpected and expected complex situations, the participants described how anticipation and planning began already during the days before surgery, when procedures and team compositions were decided. Mental models were mentioned as a strategy for preparing for patient care both ahead of future anaesthesia and when taking over a patient intraoperatively. In a previous study, RNAs also described the importance of planning for both the expected and the unexpected, using mental models and sharing a plan between RNAs and the anaesthesiologist [45]. Interruptions were often present (4.7/hr) in RNAs’ work during the immediate preparations [16]. During this phase, interruptions related to medication care and external disturbance could be interpreted as adaptations made to ensure safe care while sharing mental models with the rest of the team. Ideally, there should be time set aside for the anaesthesia team to plan ahead the requirements for safe care. Such moments are included in the WHO surgical safety checklist, but research has shown variability in the way the checklist is used [46]. The checklist provides the time necessary for sharing mental models and anticipating, which therefore should be part of the standard procedure in all operating rooms [47].

*Supporting mindful practice* was mentioned as an important strategy. At the beginning of the anaesthesia, healthcare staff could include patients in sustaining safety by informing them about the intraoperative anaesthetic care process. The importance of interaction between the patient and anaesthesia personnel was highlighted, as the situation could be very stressful to the patient, but also because the patient was seen as a team member who might detect a need for adaptation. Though communication was directed at the patient, it enabled staff to share the progress of the induction with each other [48]. Creating a peaceful atmosphere for induction was crucial in order to create cognitive space for management of any unexpected changes. However, as the preparations for induction and induction were the most task-intensive phases of anaesthesia, with multitasking and interruptions, balancing task management (e.g., medication care) and patient wellbeing in this very short timeframe was described as especially demanding. Active management of interruptions and avoiding unnecessary multitasking through planning phone use, keeping noise levels low, planning when to interrupt a colleague and asking for calm to concentrate were strategies mentioned in the interviews in regard to such situations. These strategies and tools for management of complex or safety–critical phases have been described in previous studies as well [49–52]. The so-called sterile cockpit rule prohibits unnecessary activities, including interrupting a colleague during a critical phase, such as induction or extubation [51]. Crocket et al. showed how implementation of three interventions (educating personnel on the effects of interruptions, giving the OR’s circulating nurse the responsibility to pause music prior to the arrival of the patient, and having the anaesthesiologist remind the staff in the OR to be quiet during induction) resulted in a decrease in the number of inductions including a distraction from 61 to 10% [49]. This study reinforces our previous findings that interruptions may sometimes represent adaptive capacity, as unexpected variations are an inherent part of any care process, triggering the need for the team to communicate to support preparedness and mindful practice [14, 16]. Thus, interruptions should not be categorically prohibited – but the intention and timing should be considered beforehand, possibly by agreeing within the team on when and how interrupting is ok.

Situational awareness, understanding what is going on around you by gathering information, recognizing and understanding situations or the behaviours of other team members, and anticipating the future are important for task management and teamwork [53]. As regards strategies for establishing and maintaining situational awareness for *monitoring and noticing complex situations*, the interviews captured many examples. Situational awareness can be distributed, encompassing three levels [54]. At the first level, one has to perceive elements in the environment and detect possible changes in patient care. Here, the interviewees gave many concrete examples of clues from multiple sources, such as patient monitors and the surgical team. Second, the current situation needs to be understood and, third, a projection of the future status must be made. The interviewees described how the actions, communication and mood of the surgical team provided information on possible changes which could require preparation, in addition to information gathered from observing and caring for the patient. Both professions described how certain sounds, sudden silences and smells informed them of a possible escalation of complexity and a need to react, when combined with knowledge on the specific care process phase. The ability to monitor and notice complex situations is important for adaptive capacity, reflecting the findings described in the study by Rönnberg et al., where RNAs described the management of a critical task as requiring being one step ahead, alert and using situational awareness [23]. Anaesthesia teams have a pivotal role in supporting situational awareness within the whole OR team and with other parties connected to the anaesthesia process, adding to adaptive capacity.

When *managing complex situations*, the anaesthesia team described the importance of open, timely and honest communication within the anaesthesia team as well as with the surgical team. Explicit reasoning in the form of thinking out loud and talking to the room has been shown to support the adaptation of coordination activities to meet the challenges in an emergent situation and is crucial to effective performance [55]. In our previous study, communication with all OR team members, and outside the OR, was identified as the single largest group of tasks in RNAs’ work during intraoperative anaesthesia care [16].

Clear leadership was emphasized as an important factor in any complex situation. Managing complex situations was experienced as easier when an anaesthesiologist took the lead, and any unnecessary personnel was removed from the OR. According to Larson and Holmström (2013), a sign of an excellent performance in an anaesthesiologist is being humble regarding the complexity of anaesthesia, but at the same time appearing calm and clear in critical situations and being able to present strong leadership if needed [56]. However, the interviewees also discussed that anaesthesia nurses would take charge of the anaesthesia nursing tasks in certain situations and work independently, making adaptations to protect the patient. In their study of leadership, Künzle et al. reported that leadership in anaesthesia tended to be positively correlated with team performance during non-routine and non-standardized situations. However, in routine and highly standardized situations, leadership was negatively correlated with team performance [57]. Thus, the ability to ascertain situations when care processes occur within safe boundaries, and where an anaesthesia nurse’s independent work can support and sustain high-quality teamwork is important. Moreover, the ability to notice changes and work together when a rising level of complexity threatens the boundaries of safe care may promote patient safety and increase the adaptive capacity of the team.

One key element in managing complex situations mentioned by both professions was having profound knowledge and understanding of both anaesthesia and the surgical process. Such knowledge provided the team with a shared baseline for working adaptively when needed and was described as supporting problem-solving in many ways. The connection between a shared baseline and patient safety has also been established through a survey assessing factors related to high-reliability organizational culture [58]. The survey included items such as safety culture, teamwork culture, overall patient safety grade, preoccupation with failure and adherence to shared baselines. Among these items, adherence to shared baselines and preoccupation with failure were identified as key features, both significantly correlated with overall patient safety grade [58]. Shared mental models of the situation allowed anticipating, prioritizing and understanding what was needed for future steps, but also allowed the team to collaboratively test alternative solutions calmly and without delay. Understanding of other team members’ tasks and work processes could be ensured through the basic education of OR team professionals. It could be argued that one should be acquainted with the other professions’ work processes, in addition to one’s own, in order to anticipate and predict the complexity built into everyday team work.

Team members also described how, after each complex situation, a timeout was used to walk through and learn from the management of that situation, giving and receiving feedback and checking the patient’s medications, status and position. This could be interpreted as a form of in situ learning from both critical incidents and successful management of complex and demanding situations, developing mental models ahead of future challenges and thus adding to the adaptive capacity of the team.

### Organizational prerequisites

Some of the conditions for resilient performance were created well before actual intraoperative anaesthesia by *enabling adequate levels of resources and competence* and stable teams. In another study on first-line managers in an intensive care environment, the importance of securing both enough staff and expertise for each shift was emphasized [8]. However, sustaining a unit’s resilience capacity every day requires mindful adaptations from the managers, in addition to balancing demands against capacity – two things which can never be completely aligned, due to the complex nature of the system [7, 59]. Unexpected variance and interactions will require adjustments from the frontline professionals, who in addition to following the planned protocols will also adapt to changing circumstances [59]. In the current study, the participants emphasized that managers must be aware that performance and time pressures should not exceed an anaesthesia team’s ability to adapt and work efficiently and with precision.

The importance of standardization of work processes as a way to *ensure an optimal work environment* and aid management of complex situations was mentioned by both professions. Standard operating procedures provide a core for shared baselines, establishing an important factor supporting safety in high-reliability organizations [58]. However, in a previous study from our group, managers acknowledged the need for balancing implementation and continuous updating of standard operating procedures with giving staff with deep knowledge about the clinical context room for adaptation when something unexpected happened [8].

The interviewees in this study also gave many examples of how resilient practice needs to be contextualized and that concrete strategies for maintaining safety differ from one clinical situation to another as well as between different phases of the same type of anaesthesia. They highlighted how the possibility to gain experience and practice in a focused speciality, in a relatively stable team composition, promoted deep understanding of both anaesthesia and the surgical process and possible adaptations that could be used to support safe care. As explored in previous research, NTS was said to be fundamental in managing patient safety in anaesthesia [19, 22]. The participants stated that if anaesthesia teams could gain deep understanding of NTS through training together, their ability to adapt to and manage planned and unexpected complex situations would be enhanced. The importance of simulation training to increase coordination and adaptation is supported by previous studies, especially as regards in situ simulation training [60]. The importance of experience and contextualized technical skills and NTS conflicts with the prevailing trend in healthcare to create versatile staff pools, where professionals may be distributed to a variety of units or work posts, depending on the current needs of the department and organization. This reveals an important dilemma and an example of Hollnagel’s efficiency-thoroughness trade-off principle [61]. Managers have to ensure efficiency with an adequate amount of personnel but risk losing thoroughness in the form of deep expertise, intuition and development capacity.

### Strengths and limitations

One major strength of this study was that both anaesthesia nurses and anaesthesiologists were generally quite experienced, providing rich descriptions of the phenomenon. Some of those interviewed had worked in the same department for a long time, which may have affected the variability of the data. However, we saw both saturation and variation in the responses. Furthermore, interviews were conducted in three hospitals in separate districts, to gain experiences from multiple safety cultures and organizational traditions. Individual interviews using CTA with real observation-based case scenarios added to the dependability and allowed triangulation. Individual, semi-structured interviews using CTA provided rich and relevant data. The data collection and analysis procedure was systematic – the framework method acted as a functional structure for analysis [42]. To support trustworthiness and confirmability, examples of the process from codes to themes have been presented. Credibility was supported through discussions among researchers throughout the analysis and through use of the framework method, which is suitable when researchers are situated in different locations. Also, original quotes are presented in the text, to illustrate empirically based examples. Data obtained through real-time observations were used to design case scenarios to guide parts of the CTA interviews. The scenarios were based on the findings of a previous study on safety–critical phases of intraoperative anaesthesia care [16]. Although our interview guide did not include questions regarding administration- or management-related issues, participants underlined the importance of system support. In addition, the participants provided conceptual and procedural knowledge and knowledge on performance standards.

During the planning of the study, the researcher listed factors (values, beliefs, knowledge, biases) that could affect method choice, the interview guide, the initial interviews or the data analysis. These factors were presented to and discussed in the research group, adding to the trustworthiness of the study [62]. In addition, a table of data, codes and themes has been provided to promote confirmability [63, 64].

A potential limitation of this study is that the transferability of qualitative results to other contexts is uncertain, as the results reflect the views of only 15 professionals from three different hospitals in one country and are context-dependent. However, as research conducted in other countries has indicated similar work processes and task management in anaesthesia and work contexts in the OR, our results may to some extent aid development in other countries. It could also be argued that the human experiences of responding to unexpected situations are universal. The interviewer had an RNA background, which could be seen as both a strength and a limitation. This was partially counteracted by also having a researcher without a background in anaesthesia conduct analyses of the first interviews, to compare the results in the next step. Interestingly, despite their substantial experience, participants seemed to find it challenging to verbalize the cognitive processes underlying their behaviours during everyday work process/task completion, which they perceived to be routine. This could also explain the relatively short duration of some interviews and might reflect that the strategies used for creating resilience are still very much tacit and not talked about as part of professional education and practice. We tried to address this challenge by providing the participants with visual representations of tasks, in chronological order, for both an expected and an unexpected, complex situation, based on our previous in situ observations. The length of participants’ work experience did not seem to affect interview duration.

## Conclusion

Experienced clinicians provided deep insights on what strategies support anaesthesiologists and anaesthesia nurses in managing safety in the continuously changing OR context when prompted by scenarios of complex everyday work. Adequate resources, stability in team compositions, and safe boundaries for practice based on shared baselines for recurring tasks where all viewed as important prerequisites for safe care. Although previous research has shown that management of complex situations in anaesthesia teams benefits from NTS, such as good communication, leadership and shared situational awareness, we argue that *when* and *how* to apply these skills in a specific clinical context and situation is dependent on both the right organizational prerequisites and deep expertise of the clinical context and procedure at hand. Methods such as CTA can elucidate the tacit competence of experienced staff and thus guide contextualized training and application of NTS in specific contexts and inform the design of safe perioperative work practices.

## Supplementary Information

**Additional file 1.** Interview guide.

**Additional file 2.** A study protocol / CTA.

**Additional file 3. **Themes, categories and subcathegories covered per participant.

## Data Availability

Data are available on reasonable request from the author (KO) due to them containing information that could compromise research participant privacy/consent and due to the conditions of the permission given by the Ethical Board of the University of Turku, 1/2012.

## Abbreviations

CTA: Cognitive task analysis
IQR: Interquartile range
NTS: Non-technical skills
OR: Operating room
RNA: Registered nurse anaesthetist
WAD: Work as done
WAI: Work as imagined
