# Supplementary material for: Exploring everyday work as a dynamic non-event and adaptations to manage safety in intraoperative anaesthesia care: an interview study
Source: BMC Health Serv Res. 2023 Jun 19;23:651. doi: 10.1186/s12913-023-09674-3 (PMC10278258; doi:10.1186/s12913-023-09674-3)
Supplement: Supplementary file 1 — Additional file 1. Interview guide. [file 12913_2023_9674_MOESM1_ESM.pdf]

ADDITIONAL FILE 1.

Interview guide

We are interested in how you manage routine but complex situations in your work, including multitasking and interruptions, and what (thought) strategies you have developed for handling them.

Case scenario 1. Intubation.   Case scenario 2. Maintenance

	**Questions after presentation of the case scenario**
1	Can you identify (and describe) a similar situation at your work?
2	What thoughts or feelings arise based on this case?
3	How would you handle the situation?
4	What would help you handle it?
5	What would make it hard to handle it?
6	How do you ensure that you are up-to-date with what is going on?
7	How do you anticipate the progress of the situation/procedure?
8	How might what you do differ from what a less experienced person might do in a similar situation?
9	In what ways would this be difficult for a less experienced person?
10	Can you think of an example when you have had to improvise to adapt to the demands of a situation?
11	In this situation, how did you decide what to do? What cues and strategies did you rely on?
	**Summary questions**
12	What is the most frustrating part of your work? Why?
13	What is the most rewarding part of your work? Why?
14	What would you say is the most challenging part of your work? Why?
15	Could you think of something that I have not yet asked? What should I ask?

Additional questions:
Could you explain further?
Could you give an example of that?
Would you like to add something?
Could you describe what you mean?
You mentioned this – did you really mean this or something else…?
