# Supplementary material for: Exploring everyday work as a dynamic non-event and adaptations to manage safety in intraoperative anaesthesia care: an interview study
Source: BMC Health Serv Res. 2023 Jun 19;23:651. doi: 10.1186/s12913-023-09674-3 (PMC10278258; doi:10.1186/s12913-023-09674-3)
Supplement: Supplementary file 2 — Additional file 2. A study protocol / CTA. [file 12913_2023_9674_MOESM2_ESM.pdf]

ADDITIONAL FILE 2: A Study Protocol / CTA

1. Structured observations on anaesthesia teams<br><br>Olin K, Göras C, Nilsson U, Unbeck M, Ehrenberg A, Pukk- K, et al. Mapping registered nurse anaesthetists ' intraoperative work : tasks , multitasking , interruptions and their causes , and interactions : a prospective observational study. BMJ Open. 2022;1–12.	• Data was collected partly during a structured direct observational time and motion study (WOMBAT) of operating teams (surgeons, operating room nurses and RNAs)<br>• In total 30 procedures<br>• Starting when RNA entered the OR and began to plan and prepare for the anaesthesia<br>• Lasting until the patient was transported from the OR to a recovery area.<br>• Out of the 30 procedures, two were acute and 28 electives<br>• 29 performed under general anaesthesia and one under spinal anaesthesia<br>• In 12 procedures, a nursing student was being supervised.
	• Within the OR team RNAs' had largest number of tasks, multitasking and interruptions.<br>• The proportion of time spent multitasking was highest during preparation for anaesthesia maintenance (80.2%) and before initiation of surgery.<br>• During preparation for anaesthesia induction and **in maintenance of anaesthesia**, the RNAs multitasked more than half of the time (61.9% and 63.5%, respectively).<br>• **Interruptions** occurred most frequently during **anaesthesia induction (6.2/hour**), during preoperative preparation (4.7/hour) and during preparation for anaesthesia maintenance (4.3/hour)
2. Identifying and visualizing scenarios<br>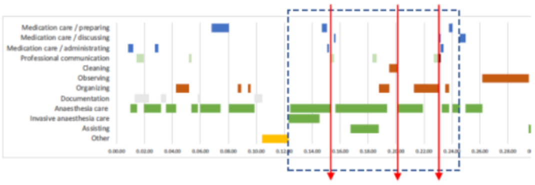	• Based on the results from observations and previous research regarding safety critical phases in intraoperative anaesthesia care two process phases (induction and maintenance) were analyzed to more detail in order to identify situations, which were normal but intensive with more than average multitasking and interruptions.<br>• Data from two such sessions were computed into a gantt - chart with excel – resulting in a visual representation of a normal but complex situation in intraoperative anaesthesia care.
3. Using scenarios when interviewing	• In the beginning of the interview, both of the visual representations were walked through by the interviewer:<br>o tasks, multitasking and interruptions identified from the gantt-chart<br>• The participants were asked, if they identified similar situations from their own work<br>• Based on their descriptions the interview-guide was used to probe the cognitive processes behind the work (example)<br>o What thoughts or feelings arise based on this case?<br>o What would help you to handle it?<br>▪ Could you explain further?<br>Add this as a flow chart
