# Supplementary material for: Exploring everyday work as a dynamic non-event and adaptations to manage safety in intraoperative anaesthesia care: an interview study
Source: BMC Health Serv Res. 2023 Jun 19;23:651. doi: 10.1186/s12913-023-09674-3 (PMC10278258; doi:10.1186/s12913-023-09674-3)
Supplement: Supplementary file 3 — Additional file 3. Themes, categories and subcathegories covered per participant. [file 12913_2023_9674_MOESM3_ESM.pdf]

ADDITIONAL FILE 3.

Sustaining safety during complex situations	Anaesthesiologists (ID)	Anaesthesia nurses (ID)
**Strategies**		
Being prepared		
Planning for the anaesthesia during the preceding day		3, 6
Preparing for possible emergencies	3, 4,	1, 2, 3, 4, 5, 7
Using mental models to anticipate events	4, 5	
Deep understanding of both anaesthesia and the surgical process	1, 3, 5, 6	1, 2, 3, 4, 5, 6
Supporting mindful practices		
Creating a peaceful atmosphere for the patient		1, 2, 3
Keeping noise levels low, planning the usage of phones and when to interrupt a colleague	1, 2, 3, 4, 6	1, 5, 7, 8
Using memory aids to focus on the primary task		1, 2, 3, 4, 6
Monitoring and noticing complex situations		
Working adaptively inside the safe boundaries		2, 5, 6
Monitoring the patient may provide clues regarding a change	1, 2, 3	2, 3, 5, 7, 8
Observing the mood and actions of the surgical team	1, 2, 3, 4, 6	1, 3, 6, 8
Managing complex situations		
Prioritizing, knowing the next steps and testing alternative solutions calmly, without delay	1, 2, 3, 4, 5	1, 2, 3, 5, 7, 9
Clear and undivided leadership		1, 2, 3, 4, 5, 6, 9
Open, timely and honest communication	1, 5, 6	2, 4, 5, 7, 8, 9
A timeout after a complex situation for checks and feedback	5	2, 6
**Organizational prerequisites**		
Enabling adequate levels of resources and competence		
Adequate amount of personnel and stable teams	1, 2, 3, 4, 6	1, 2, 3, 4, 6, 9
Simulation training promotes the ability to react and adapt	5, 6	3, 6
The personal wellbeing of the anaesthesia professionals	1, 3	7
Performance and time pressures should be manageable	2, 3	4, 5, 7, 9
Ensuring an optimal work environment		
Standardization of processes and work environment	1, 2, 3, 4, 5, 6	1, 2, 3, 6, 8, 9
Suitable and functional electronic patient records, equipment and appliances	3, 5	1, 4, 7
